# Sleep pattern and practice among adolescents school children in Nigerian secondary schools

**DOI:** 10.11604/pamj.2014.19.313.4603

**Published:** 2014-11-21

**Authors:** Josephat Chinawa Maduabuchi, Herbert Anayo Obu, Barthlomew Friday Chukwu, Ann Ebele Aronu, Pius Chukwuka Manyike, Awoere Tamunosiki Chinawa

**Affiliations:** 1 Department of Paediatrics, University of Nigeria/Teaching Hospital, Enugu, Nigeria

**Keywords:** Adolescents, sleep pattern, secondary school

## Abstract

**Introduction:**

Some adolescents may have sleep disorder at some point during adolescence. Determining the pattern and practice of sleep among adolescents could be useful to establish a lasting sleep hygiene program among adolescents. The objectives of this study are to describe sleep pattern and practice among adolescent in Nigerian secondary schools.

**Methods:**

Sleep habits were investigated using a random sampling of adolescents from secondary schools from February to April 2013. A self-administered questionnaire was developed based on the Diagnostic and Statistical Manual of Mental Disorders (DSM) IV criteria. Epworth Daytime Sleepiness Scale and Pittsburgh Sleep Quality Index (PSQI) were used.

**Results:**

A total of 443 subjects, comprising 263 (59.4%) females and 180 (40.6%) males completed the questionnaire. The mean duration of night sleep of the subjects during weekday was 7.84 (1.9) hours and 8.65 (2.07) hours during the weekend. 22.8% (101/443) had abnormal sleep onset latency (< 5 minutes and > 30 minutes). The gender of the subjects did not influence the sleep onset latency (χ^2^ = 32.89, p= 0.57). Twenty six (5.9%)of the subjects reported difficulty falling asleep.

**Conclusion:**

Adolescents have varying degrees of sleeping practice and hygiene.

## Introduction

Sleep problem usually entails a sleep pattern that is unsatisfactory or cause of concern to the parent, child or physician. Children present to primary care physicians or pediatricians with mainly three types of sleep related problems-first group has disorders of initiating and maintaining sleep (dyssomnias); second category (hypersomnias) is characterized by excessive sleepiness and third section represents abnormal activity or behavior during sleep, also classified as parasomnias [1]. The average person spends approximately one third of his or her life sleeping [2]. Although sleep comprises such a significant portion of a child's day, sleep disturbances are often overlooked by healthcare practitioners. Bedtimes and sleep start times were earliest and time in bed and sleep period times were longest for 12-month-old children. Rise time, sleep end time, and nocturnal sleep minutes did not differ across age groups. Actigraphic estimates indicated that children aged 1 to 5 years slept an average of 8.7 hours at night [3]. Throughout childhood, children typically get about 10 hours of sleep a night. This drops significantly at adolescence, but less for biological reasons than for socio-cultural reasons. Sleep researchers studying the optimal sleep periods of teenagers have found that when the sleep-wake cycle is studied in the laboratory under controlled conditions (e.g., removing clocks and lighting cues), teenagers typically sleep 9 hours a night. In the real world-especially during the school year-very few teenagers get this much sleep and thus are constantly coping with sleep debt to a greater or lesser degree [4–6].

Behavioral sleep problems defined as sleep problems that do not have a physiological aetiology, but rather a behavioral are reported in between 20% - 40% of children and adolescents [7]. These sleep disorders are categorized as behavioral Insomnia of Childhood (BIC) in the International Classification of Sleep Disorders. These can result in short sleep duration and poor quality sleep and can have wide ranging effects on mental and physical health, cognitive and social functioning and development in infants, pre-scholars; school aged children and adolescents [7]. Epidemiological studies performed in Western Europe, the USA, and Japan have reported a prevalence of insomnia-related symptoms ranging from 10% to 48%. [7–9]. The differences in the prevalence of insomnia among the different studies is not only due to cultural differences, but is also attributed to how one defines “insomnia”. Sleep disorders are associated with an increased prevalence of various somatic and/or psychiatric disorders as well as social problems [10]. Adolescent sleep patterns deserve particular attention because of their potential to affect school performance. Adolescents typically get significantly less sleep than younger children, not because they need less sleep but because their schedule and biorhythms impede adequate sleep [11]. Carskadon and colleagues studying the optimal sleep periods of adolescents, found that under controlled conditions (e.g., with no clocks and lighting cues), adolescents typically sleep nine hours a night [11]. About a quarter of adolescent need more sleep than they previously had, and 10% of them complained of difficulty falling asleep. Adolescents reporting sleep problems showed more anxious, depressed, inattentive, and conduct disorder behaviors than those who had no sleep problems [12].

There exist a connection between socioeconomic class and sleep habits among adolescents, for instance Kahn et el [13] noted that distorted sleep pattern are commoner in adolescents whose parents are divorced or from a low socioeconomic class. Little is known about sleep patterns and problems in adolescents in this vicinity. Data from this part of the world is lacking and sleep habits among adolescents have gone unreported. This study was thus designed to examine sleep/wake practice of sleep among adolescents in Nigeria. It is hoped that this will add to the body of knowledge available on these disorders and the findings of this study could form the template for intervention strategies in helping reduce this social aberrations.

## Methods

### Study Area

The study was conducted in secondary schools from the city of Enugu in South-Eastern Nigeria. Enugu is an urban area, It lies in the rainforest zone with two major seasons (Rainy and Dry) and a temperature of about 28-33oc. It has a population of about 500,000 people according to the National population census [14].

### Study Population

This was a prospective study enrolling adolescent students aged 13-19 years in secondary schools located in south east Nigeria selected by random sampling. This study was carried out from February to April 2013 using convenient sampling method.

### Instrumental tools used in the study

**Pittsburg Quality of Sleep Index (PQSI)** [15]: It is a self report instrument to assess the quality of sleep. The questions are framed into 4 points and it analyzes factors such as sleep quality, sleep latency, sleep duration, sleep efficiency, sleep disturbance and use of sleep medication. We modified this questionnaire to fit into our environment. We added demographic characteristics.

**Epworth Daytime Sleepiness Scale (EDSS)** [16]: It is a scale intended to measure daytime sleepiness that is measured by use of a very short questionnaire. This can be helpful in diagnosing sleep disorders. It was introduced in 1991 by Dr. Murray Johns of Epworth Hospital in Melbourne, Australia. Daytime sleepiness (DTS) was studied using the Epworth daytime Sleepiness Scale (EDSS) which is a reliable validated sleep questionnaire to measure DTS. It consists of eight items including different situation and activities of everyday life. The version and section of DSM used is DSM-IV section 307.42 -307.47 (dyssomnias and parasomnias). In this study, Sleep hygiene or good sleeping practices are defined as a variety of different practices that are necessary to have normal, quality nighttime sleep and full daytime alertness [9]. Confidentiality was assured to all students who were asked to volunteer and none were reimbursed. Students who were willing to participate were given a brief description about the study and its objectives. Objective of this study was to describe sleep practices and pattern among adolescents in two Nigerian secondary schools. Students who gave consent to participate in this study were included in the study while adolescents who were currently using sedative medications or narcotics for any acute or chronic medical condition were excluded.

### Ethical Considerations

Ethical clearance for the study was sought from the Research and Ethical Committee of the University of Nigeria Teaching Hospital Ituku Ozalla. However since this work was done in a secondary school and not in the Hospital, we did not bother to finally procure the consent from the hospital. Verbal Consent was obtained from the adolescents, caretakers and teachers after they had been told that their participation was completely voluntary in nature, and that they could discontinue their involvement at any time. Anonymity and confidentiality of responses was also conveyed. We did not obtain a written consent because we wanted the work to be concluded within a short period, thus avoiding the protocols involved in getting a signed written consent. The procedure was accepted by the Head teacher and as such we did not document the procedures. Since the work was not executed in the hospital, the ethical committee did not bother to approve or reject the approval, as far as consent was granted by the head teacher. We did not obtain informed consent from the next of kin, caretakers, or guardians, since we believe that the head teacher has already granted us consent.

### Data Analysis

All data were coded, entered, and then analyzed using the Statistical Package for Social Sciences program (SPSS), version 16. Descriptive results were expressed as frequency, percentage, and mean ± S.D. P-values < 0.05 were accepted as statistically significant. Pearson chi-square was used to test for significant relationships between categorical variables. Non-parametric Spearman Rank Order was used to test for correlation between continuous and ordinal variables. A difference in means between groups was carried out using student's t- test.

## Results

### Demography

A total of 443 subjects, comprising 263 (59.4%) females and 180 (40.6%) males completed the questionnaire. The mean age of subjects was 15.79 (1.40) years. The mean age for females was 15.75 (1.42) and that of males was 15.85 (1.34). The age of both sexes was well matched (t= 0.75, p= 0.45). Subjects socioeconomic distribution showed that 183 (41.3%), 144 (32.5%) and 116 (26.2%) belonged to the high, middle and low socioeconomic class respectively. There was no significant difference in the gender and socioeconomic class distribution of the subjects. Table 1 shows the gender and social class distribution of the subjects.

**Table 1:** Gender and social class distribution of subjects

Gender	High	Social class Middle	Low	Total
Female	113	85	65	263
Male	70	59	51	180
Total	183	144	116	443

χ^2^ = 0.97, p= 0.62

### Sleep habits

Result of the sleep onset latency of the subjects showed that 59 (13.3%) had sleep onset latency of less than 5 minutes, 41.8% (185/443) had latency of 5-10 minutes, 35.4% (157) had latency of 11-30 minutes while 9.5% (42) had sleep onset latency of more than 30 minutes. This indicates that 22.8% (101/443) had abnormal sleep onset latency (< 5 minutes and > 30 minutes). The gender of the subjects did not influence the sleep onset latency (χ^2^ = 32.89, p= 0.57). The mean duration of night sleep of the subjects during weekday was 7.84 (1.9) hours and 8.65 (2.07) hours during the weekend. There was a significant difference in the mean duration of night sleep during the weekday and weekend (t = 6.12, p<0.001). There was no significant difference in the mean duration of night sleep between the males and females either during the weekday or weekend (p=0.51 and 0.88 respectively). On nocturnal awakening frequency, the subjects reported as follows: 85.6% (380/443) had nocturnal awakening frequency of 1-2 times per night, 12.9% (57/443) had frequency of 3-4 times per night and 1.4% (6/443) had frequency of more than 4 times per night.

### Paranosmia (unusual sleep behaviors)

Some of the subjects did display unusual sleep behaviours such as sleep walking, sleep talking, bruxism or act out dreams. Among the 443 respondents, 28 (6.3%) did sleep walk, 15.1% sleep talk, 7.6% grind their teeth (bruxism), 16.5% had restless leg syndrome while 26.4% act out their dreams. There was no correlation between unusual sleep behaviours and sleep latency or night awakening frequency. Table 2 shows the frequency of unusual sleep behaviors among the subjects.

**Table 2:** Frequency of unusual sleep behaviors (paranosmia) of the subjects

Unusual sleep behavior	Frequency	Percentage%
Sleep walking	28	6.3
Sleep talking	57	14.1
Bruxism	34	7.6
Restless leg syndrome	73	16.5
Act out dreams	117	26.4

### Sleep problems

Twenty six (5.9%)of the subjects reported difficulty falling asleep while 2.0% and 2.9% reported restless sleep and morning headache respectively. Nine subjects (2.0%) did always experience uncomfortable sensation in their legs during sleep. About 6.6% did hallucinate while falling or awakening from sleep, 2.0% did report of libido/erectile dysfunction while sleeping while 3.8% reported feeling of panic on awakening from sleep. On the question of how likely one will fall asleep in some usual ways of life, 14.4% (64/443) did respond that they have high chance of falling asleep while sitting and reading. Also about 7.4% have a high chance of falling asleep while sitting and talking to someone. Twelve (2.7%) of the subjects admitted they have a high chance of sleeping while driving.

## Discussion

Adolescents go to bed earlier on weekdays than on weekends, with a total night time sleep of 7.84 hours and 8.65 hours on weekdays and on weekends respectively. Ideally adolescents need 9 hours and 15 minutes of sleep, children need 10 hours and adults need 8 1/4 hours [17]. This is less than what we obtained in our study. The decrease in total sleep duration in adolescence reflects a shift in sleep/wake patterns that begins during puberty and is marked by later bedtimes in conjunction with earlier rise times. [18, 19]. Other reasons noted for this short sleep period among adolescents could be due to early school start time, inability to fall asleep until late at night, their boisterous and energetic social life and homework to do. This shortened sleeping hours among adolescent was also reported by Kutluhan et al [18] who obtained a sleeping time of 7hrs and 8hrs on weekdays and on weekends respectively.

In keeping with findings in Western countries [20] and several studies in Asian countries [21–23] we noted that adolescents went to bed later and woke up later in the morning on weekends than on weekdays. The longer sleep duration on weekends, compared with weeknights, suggests that adolescents are relatively sleep deprived on weekdays. Longer weekend sleep duration may reflect their actual sleep need or may be an indicator of “catch-up” sleep to compensate for sleep insufficiency on weekdays. [24] It is interesting to note from this study, that the average difference between school day and weekend rise times, referred to as weekend rise time delay is 1 hour 6 minutes. This was found to be significant. This weekend rise time delay was also corroborated by Crowley [25] and colleagues who noted an average difference of 1.5hours. Likewise, the difference between school day and weekend bedtimes (i.e., weekend bedtime delay) averages 1.5 hours. Changes in both circadian and homeostatic processes during puberty appear to be an explanation to this time difference. In laboratory studies, pubertal stage has been found to be positively associated with later circadian timing and slower accumulation of homeostatic sleep pressure [26, 27]. Thus, as children age, each night of sleep becomes less dependent on the quality and quantity of the prior night's sleep.

About a quarter of adolescents studied reported abnormal sleep latency. LaBerge [28] et al in their sleep study among 19 adolescents in Canada documented a sleep latency of between 8.5-14.7minutes. Though this may vary from that obtained from other study. The abnormal sleeping latency obtained in this study may be due to poor feeding habit and fear of not covering the day's work especially during exams. Other reasons for these differences could be due to differences in geographical locations and religio- cultural differences. For instance, in Enugu, there are long school hours on weekdays, extra mural classes are also organized for these students after normal school hours. The adolescents also have a very busy, highly social weekend schedules and rampant religious activities even in weekdays. All these have impact on sleeping habits of adolescents. This data was also collected in the rainy season over a three months period. This could also affect sleep habits of the adolescents studied. We noted no significant difference between mean duration of night sleep on weekdays and weekends when compared to gender. This is also in keeping with the work of Morrison and colleagues [29] and Sochat [30] noted no gender difference in sleep pattern.

Majority of the adolescent studied had nocturnal awakening frequency of 1-2 times per night. This is similar to that of Ravi [31] et al who obtained nocturnal awakening frequency of 0.8-2.4 times per night. We reported unusual sleep behaviors, such as sleep walking, sleep talking, bruxism, restless leg syndrome or act out dreams. This is close to that obtained by LaBerge [32] and colleagues who also noted similar cases. It is depicted that some parasomnias are strongly associated with each other, such as sleepwalking, night terrors, and somniloquy (sleep talking), and may represent 1 type of parasomnias, whereas leg restlessness, body rocking, sleep bruxism, and enuresis would each represent unrelated clinical entities without specific association to any sleep stage. [32] Social class difference did not influence the mean duration of sleep among the adolescents in this present study. There exists a connection between socioeconomic class and sleep habits among adolescents. Contrariwise, Kahn et el [13] noted that distorted sleep pattern are commoner in adolescents whose parents are divorced or from a low socioeconomic class.

## Conclusion

"Adolescents have varying degrees of sleeping practice and hygiene". We did not do sleep studies like Polysomnogram, Maintenance of Wakefulness Test due to lack of facility.
